# Defervescent dengue patients might be a potential source of infection for vector mosquitoes

**DOI:** 10.1186/s40249-020-0631-8

**Published:** 2020-03-02

**Authors:** Ye Xu, Ling-Zhai Zhao, Ya-Zhou Xu, Jin-Bao Gu, Kun Wu, Zhi-Qiang Peng, Xiao-Hong Zhou, Fu-Chun Zhang, Xiao-Guang Chen

**Affiliations:** 1 Department of Pathogen Biology, School of Public Health, Southern Medical University, Guangzhou, China; 2 Guangzhou Eighth People’s Hospital, Guangzhou Medical University, Guangzhou, China; 3 Guangdong Provincial Center of Disease Control and Prevention, Guangzhou, China

**Keywords:** Dengue fever, Dengue virus, Vector, *Aedes aegypti*, *Aedes albopictus*

## Abstract

**Background:**

Dengue is a re-emerging public health problem and mosquito-borne infectious disease that is transmitted mainly by *Aedes aegypti* and *Ae. albopictus*. Early diagnosis, isolation, and treatment of patients are critical steps for dengue epidemic control, especially to prevent secondary transmission of dengue virus (DENV). However, little is known about defervescent dengue patients as a source of infection.

**Methods:**

This case study describes 1268 dengue patients hospitalized at Guangzhou Eighth People’s Hospital from June 2013 to December 2014. The viral loads of each individual were measured using real-time reverse transcription-polymerase chain reaction. *Ae. aegypti* and *Ae. albopictus* were exposed to blood meal with gradated dengue viral loads to characterize the relationship between viremia in dengue patients and the vector competence of vector mosquitoes.

**Results:**

The viral numbers in the blood were measured, ranging from 10^8^ to 10^3^ copies/ml from day 1 to day 12 after fever onset. Vector competence analysis of *Ae. aegypti* and *Ae. albopictus* indicated that viremia > 10^4^ copies/ml can still infect vector mosquitoes, which implied that the defervescent dengue patients might be a source of infection.

**Conclusions:**

The results of this study indicate that some defervescent dengue patients still have sufficient viral load to infect vector mosquitoes. Therefore, the protection against mosquito biting for these people should be extended to prevent secondary transmission events.

## Background

Dengue is a mosquito-borne infectious disease and is transmitted mainly by *Aedes aegypti* and *Ae. albopictus*. Early diagnosis, isolation, and treatment of patients are critical steps for dengue epidemic control, especially to prevent secondary transmission of dengue virus (DENV) [1, 2]. The host and virus variables are associated with DENV transmission from symptomatic dengue cases to vector mosquitoes [3]. Moreover, at a given level of viremia, DENV-infected people with no detectable symptoms or not yet exhibiting symptoms are significantly more infectious to mosquitoes than people with symptomatic infections [4]. At present, the discharge criteria for dengue patients set by the WHO and China are as follows: no fever for 24–48 h, improvement in clinical status, increasing trend of platelet count, and stable hematocrit without intravenous fluids [5, 6]. Is it possible for defervescent dengue patients to be a source of infection? What is the changing role of viremias in dengue patients after fever onset? Is there any minimum DENV copy number required to infect vector mosquitoes? To answer these questions, we analyzed the viremias in 1268 dengue patients ranging from day 1 to day 12 after fever onset and tested the infection rates of various viral loads of DENV in blood meals on vector mosquitoes.

## Methods

### Study design

Dengue patients hospitalized at Guangzhou Eighth People’s Hospital from June 2013 to December 2014 were included in this study. The diagnosis of dengue patients was performed in accordance with the 2009 WHO guidelines for dengue diagnosis, treatment, prevention, and control. Inclusion criteria were patients positive for DENV RNA, as determined by reverse transcription-polymerase chain reaction (RT-PCR), and nonstructural protein 1 (NS1) or virus-specific IgM antibody, as determined by enzyme-linked immunosorbent assay (ELISA) test.

### Sample size determination

Blood samples of 3–5 ml were collected, and serum was separated by centrifugation. In total, 1268 cases of dengue patients hospitalized at Guangzhou Eighth People’s Hospital from June 2013 to December 2014 were included in this study. All infections belonged to serotype I. The median age was 48 years (range 17–90 years). The male/female ratio was 1∶0.83 (693 male cases and 575 female cases). A total of 1152 cases were classified as dengue fever, and 116 were severe dengue. Overall, 1955 serum samples were collected for viral load detection (some patients provided several serum samples at different time points).

### Viral load quantification

DENV loads were determined using the DENV1–4 One-Step Real-Time RT-PCR Kit (DaAn Gene, Guangzhou, China) following the manufacturer’s instructions. Each sample was tested at least in duplicate, and each experiment was performed twice and included negative and positive controls. The limit of detection was 100 copies/ml.

### *Ae. aegypti* and *Ae. albopictus* treated with blood meals with gradated dengue viral loads

We used artificial blood meals with graded dengue viral loads to explore the relationship between viremia in dengue patients and the infection of vector mosquitoes, *Ae. aegypti* and *Ae. albopictus,* according to the methods described in previous studies [7–9]. In brief, infectious blood meals were prepared by mixing defibrinated sheep blood (Solarbio, Beijing, China) with gradated fresh DENV viral suspension at a ratio of 1∶2. For treatment, blood meals with a tenfold dilution series of viral load (10^10^ to 10^4^ copies/ml) were used. Each blood meal was warmed to 37 °C and transferred into a Hemotek blood feeding system (Discovery Workshops, Blackburn, UK) for mosquito feeding. Five days after eclosion, *Ae. aegypti* and *Ae. albopictus* females were used. After exposure for 30 min, a total of 2486 *Ae. albopictus* and 664 *Ae. aegypti* were anesthetized with ether, and fully engorged females were selected for further testing. The infection rates of *Ae. albopictus* on the 4th day post infection (dpi) (*n* = 1222) and the 14th dpi (*n* = 1264) and *Ae. aegypti* on the 4th dpi (*n* = 371) and 14th dpi (*n* = 293) after blood feeding were determined by PCR with DENV-specific primers. Experiments were performed in accordance with standard procedures in a Biosafety Level 2 laboratory.

## Results

### Viral load throughout illness course

**Fig. 1:**
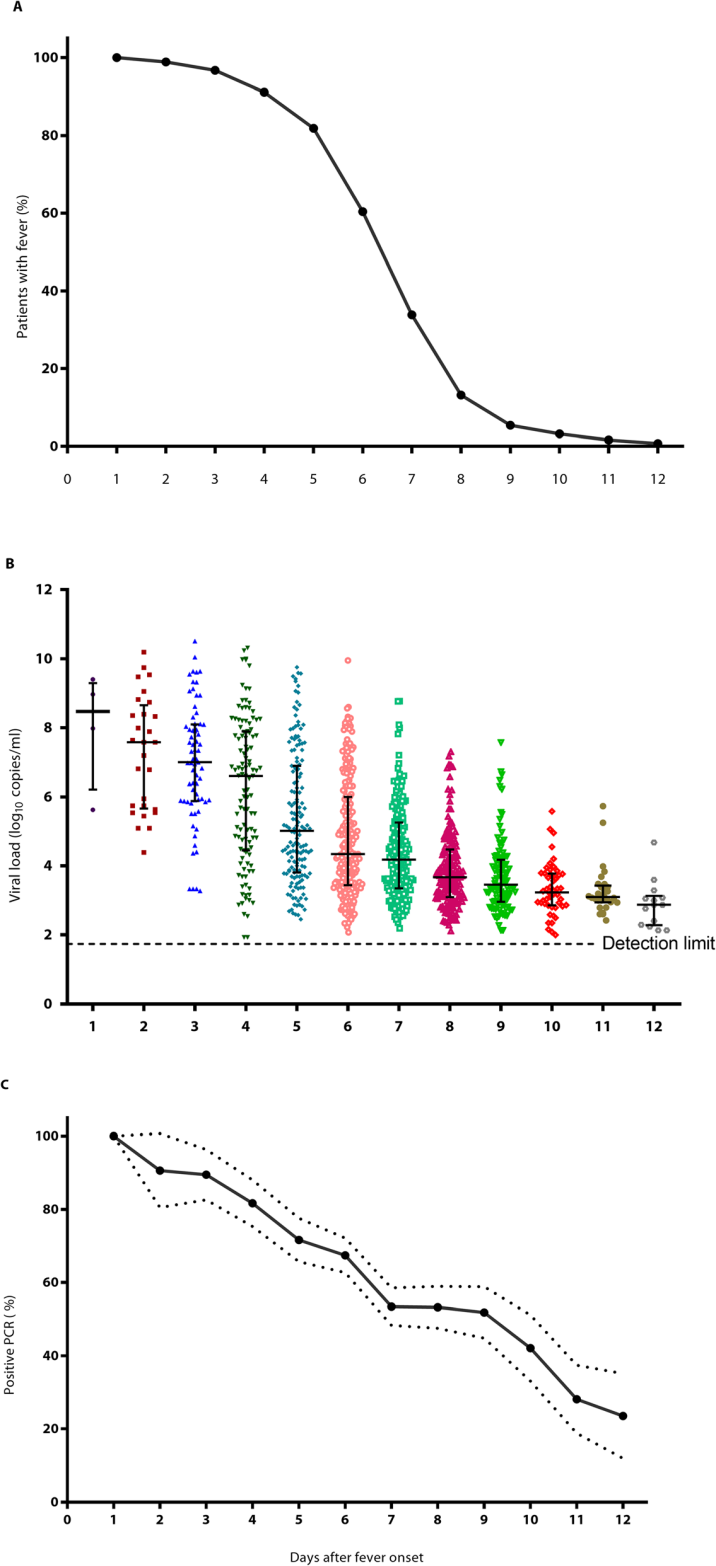
Characterization of 1268 dengue patients after fever onset. **a** Percentage with fever. **b** Viral load. Each dot represents the virus copies of individual patients. Error bars indicate SDs. **c** Percentage of positive PCR. reverse transcription-polymerase chain reaction (RT-PCR)

The kinetics of the percentage of patients with fever throughout the illness course, viral load of different patients throughout the illness course, and percentage of patients with positivity for DENV RNA are summarized in Fig. 1. All patients had fever at the onset of disease, which lasted 5–6 days in most cases. The viral load gradually decreased as patients recovered from the disease. The percentage of patients who were positive for DENV RNA also declined from 100% on day 1 to 23.53% on day 12 after disease onset. Dengue viremia kinetics in dengue patients suggested that the maximum observed viral load reached an average of 10^8^ copies/ml on the first day of fever. Then, the viral load gradually decreased over the days with fever. The viral load decreased to an average of 10^3^ copies/ml on day 12 after fever.

### Vector competence under gradated viral loads of *Ae. aegypti* and *Ae. albopictus*

The infection rates in both *Ae. aegypti* and *Ae. albopictus* decreased as the viral load decreased in the blood meal. For *Ae. aegypti*, the infection rates reached 100% when the viral load in the blood meal was 10^10^ copies/ml and 10^9^ copies/ml on the 14th dpi. When the viral loads were below 10^6^ copies/ml, the infection rates became 0% on the 4th dpi. However, the viral load of 10^4^ copies/ml could still infect *Ae. aegypti* with an average infection rate of 9.4% on the 14th dpi after the blood meal. For *Ae. albopictus*, when the viral load of a blood meal was 10^10^ copies/ml, both the infection rates on the 4th dpi and 14th dpi were more than 95%. However, when the viral loads were below 10^5^ copies/ml, the infection rates became 0% on either the 4th dpi or the 14th dpi after the blood meal. These results indicated that it is possible to infect *Ae. aegypti* and *Ae. albopictus* when the viral copy number in viremia was greater than 10^4^ copies/ml or 10^6^ copies/ml, respectively.

## Discussion

In this study, the infection rates on the 4th dpi and 14th dpi of both *Ae. aegypti* and *Ae. albopictus* were measured. On the 4th dpi, DENV began to spread to other organizations, which indicated an early infectious status after infection. On the 14th dpi, DENV was detected in the midgut, salivary grand, ovary, and head, which indicated a stable infectious status after infection. To set a rigid gradient virus titer and simulate the infection events, we used artificial blood feeding. Although artificial blood feeding could not present the same condition as natural human blood, it could obtain controllable treatments and reliable results and has been used in many studies for infection assays [7].

**Fig. 2:**
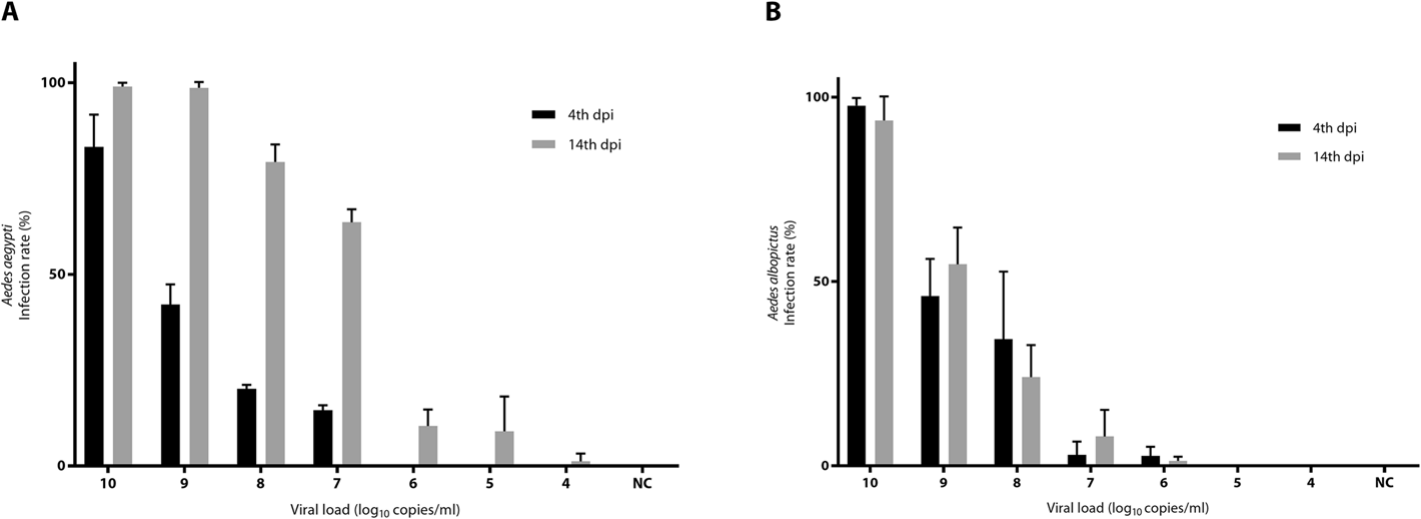
Infection rates of **a**: *Ae. aegypti* and **b**: *Ae. albopictus* fed on blood with various dengue viral copy numbers. The infection rates were measured 4th (black) and 14th dpi (gray) after the blood meal. Error bars indicate SDs. NC stands for negative control without virus in the blood meal. All experiments were repeated three times. Dpi: Day post infection

Most of the 1268 dengue patients exhibited fever for 5–6 days and were discharged 1–2 days after defervescence. Our results showed that viral loads in 1268 dengue patients gradually decreased from 10^8^ to 10^3^ copies/ml after fever onset (Fig. 1), and vector mosquitoes, *Ae. aegypti* and *Ae. albopictus*, can still be infected when the viral copy number is greater than 10^4^ copies/ml and 10^6^ copies/ml in the blood meal, respectively (Fig. 2). However, the viral load of 70.4% (112/159) of patients on day 5 after fever onset was above 10^4^ copies/ml, which means that the viremia in these patients could be an infectious source of dengue virus. On day 8 after fever onset, approximately 85% of patients did not have fever, but the viral load of 35.8% (58/162) of patients was above 10^4^ copies/ml, which is sufficient to infect vector mosquitoes. On day 12 after fever onset, there were no patients with fever, and only 7% (1/14) had a viral load above 10^4^ copies/ml. According to our results, a virus load below 10^6^ copies/ml could not infect *Ae. aegypti* on the 4th dpi and below 10^4^ copies/ml on the 14th dpi. A virus load below 10^5^ copies/ml could not infect *Ae. albopictus* on the 4th dpi and the 14th dpi. In the field, *Ae. aegypti* and *Ae. albopictus* can survive for more than one month. The viral load of a few patients was still above 10^4^ copies/ml on day 12 after fever onset. Currently, the discharge criteria for dengue patients, as set by the WHO and China, are as follows: no fever for 24–48 h, improvement in clinical status, increasing trend of platelet count, and stable hematocrit without intravenous fluids [5, 6]. These criteria do not guarantee that viral loads are insufficient to infect vector mosquitoes. This possibility would provide an opportunity to infect vectors and enable a secondary epidemic.

Our results are highly reliable and consistent with previous studies describing the various viral loads in dengue patients at different clinical stages and demonstrating that a certain number of DENV copies are required for the infection of vector mosquitoes [3, 4, 10]. These results implied that the viremia in patients after defervescence might be a source of infection, but this possibility becomes increasingly faint as more time passes without fever.

## Conclusions

The results of this study indicate that some defervescent dengue patients still have sufficient viral load to infect vector mosquitoes. Therefore, the protection against mosquito biting for these patients should be extended, by seven days after defervescence, for example, to reduce the potential infectious sources of dengue.

## Data Availability

No data and material are available for sharing.

## Abbreviations

DENV: Dengue virus
ELISA: Enzyme-linked immunosorbent assay
NS1: Nonstructural protein 1
RT-PCR: Reverse transcription-polymerase chain reaction
WHO: World Health Organization
